# Chemosensitivity profiling of osteosarcoma tumour cell lines identifies a model of BRCAness

**DOI:** 10.1038/s41598-018-29043-z

**Published:** 2018-07-13

**Authors:** Harriett Holme, Aditi Gulati, Rachel Brough, Emmy D. G. Fleuren, Ilirjana Bajrami, James Campbell, Irene Y. Chong, Sara Costa-Cabral, Richard Elliott, Tim Fenton, Jessica Frankum, Samuel E. Jones, Malini Menon, Rowan Miller, Helen N. Pemberton, Sophie Postel-Vinay, Rumana Rafiq, Joanna L. Selfe, Alex von Kriegsheim, Amaya Garcia Munoz, Javier Rodriguez, Janet Shipley, Winette T. A. van der Graaf, Chris T. Williamson, Colm J. Ryan, Stephen Pettitt, Alan Ashworth, Sandra J. Strauss, Christopher J. Lord

**Affiliations:** 1 The CRUK Gene Function Laboratory and Breast Cancer Now Toby Robins Research Centre, The Institute of Cancer Research, London, SW3 6JB UK; 2 UCL Cancer Institute, University College London, London, WC1E 6DD UK; 3 Clinical and Translational Sarcoma Research, The Institute of Cancer Research, London, SM2 5NG UK; 4 The Royal Marsden NHS Foundation Trust, Fulham Road, London, SW3 6JJ UK; 5 School of Biosciences, University of Kent, Canterbury, Kent CT2 7NJ UK; 6 Sarcoma Molecular Pathology Laboratory, The Institute of Cancer Research, London, SM2 5NG UK; 7 Edinburgh Cancer Research Centre, IGMM, University of Edinburgh, Edinburgh, EH4 2XR UK; 8 Systems Biology Ireland, University College Dublin, Dublin 4, Ireland; 9 Present Address: UCSF Helen Diller Family Comprehensive Cancer Centre, San Francisco, California 94158 USA

## Abstract

Osteosarcoma (OS) is an aggressive sarcoma, where novel treatment approaches are required. Genomic studies suggest that a subset of OS, including OS tumour cell lines (TCLs), exhibit genomic loss of heterozygosity (LOH) patterns reminiscent of BRCA1 or BRCA2 mutant tumours. This raises the possibility that PARP inhibitors (PARPi), used to treat BRCA1/2 mutant cancers, could be used to target OS. Using high-throughput drug sensitivity screening we generated chemosensitivity profiles for 79 small molecule inhibitors, including three clinical PARPi. Drug screening was performed in 88 tumour cell lines, including 18 OS TCLs. This identified known sensitivity effects in OS TCLs, such as sensitivity to FGFR inhibitors. When compared to BRCA1/2 mutant TCLs, OS TCLs, with the exception of LM7, were PARPi resistant, including those with previously determined BRCAness LoH profiles. Post-screen validation experiments confirmed PARPi sensitivity in LM7 cells as well as a defect in the ability to form nuclear RAD51 foci in response to DNA damage. LM7 provides one OS model for the study of PARPi sensitivity through a potential defect in RAD51-mediated DNA repair. The drug sensitivity dataset we generated in 88 TCLs could also serve as a resource for the study of drug sensitivity effects in OS.

## Introduction

Whilst there have been a number of successes in developing targeted approaches to carcinomas, for many patients diagnosed with hard to treat sarcomas, radical surgery and chemotherapy regimens still represent the standard of care, and survival rates have not significantly changed for some time. This is particularly true for patients diagnosed with osteosarcoma (OS), where there has been little improvement in overall survival in the past 20 years, with five year survival remaining approximately 70% for young patients with localised disease^1,2^. For those with metastatic OS at diagnosis, outcome is worse, with only 20–30% surviving for more than five years^3^ and is even more dismal for those with recurrent disease^4^. There is a paucity of early phase clinical trials available for OS patients and therefore, new or repositioned agents are needed to improve the outcome of patients. With this in mind, recent work has suggested that a significant proportion of OS, perhaps as high as 80%, exhibit genomic features reminiscent of a ‘BRCAness’ phenotype^5^. BRCAness describes tumours that possess histopathological and molecular characteristics similar to those found in patients with germ-line *BRCA1* or *BRCA2* gene defects, including a distinctive DNA repair defect arising from loss of Homologous Recombination (HR) and drug sensitivity effects associated with this HR defect, including exquisite PARP inhibitor sensitivity^6^. Using an analysis of whole-exome sequencing from 31 treatment naive osteosarcoma tumour samples, Kovac *et al*. identified large-scale genome instability signatures characteristic of BRCA1/2-deficient tumours and recurrent mutations in tumour suppressor genes associated with homologous recombination and related DNA repair processes, including *FANCA BRCA2* and *ATM*^5^. A subsequent analysis of OS tumour cell lines by Engert, Kovac *et al*.^7^ suggested that two OS tumour cell line models (MG63 and ZK58) with genomic loss of heterozygosity characteristics somewhat reminiscent of BRCAness, exhibited sub-micromolar IC_50_ sensitivity to the clinical PARP inhibitor talazoparib (MG63 = 0.448 uM, ZK58 = 0.115 uM). However, whether the scale of PARP inhibitor sensitivity seen in OS models resembles that in *BRCA* gene mutant tumour cell lines was unclear.

Despite the need to identify novel targeted approaches in OS and the availability of a number of relatively well characterised tumour cell lines derived from these tumours, there is a limited amount of publically available information describing the small molecule sensitivity of these models. Such information could provide the starting point for discovering novel treatment approaches for this particular sarcoma subtype. With this in mind, we describe here as a resource, a dataset detailing the chemosensitivity of 18 OS tumour cell lines, and an additional 70 tumour cell lines derived from non-OS cancer histologies, to 79 small molecule inhibitors used in the treatment of cancer or in late stage development. As part of this resource, we describe the sensitivity of OS tumour cell lines to three chemically distinct PARP inhibitors, comparing their sensitivity to *BRCA1* mutant tumour cell lines and to a tumour cell line model with a CRISPR-Cas9 mutagenesis-engineered PARP inhibitor resistance-causing revertant mutation in *BRCA1*. We find that that the majority of OS tumour cell lines, with the exception of LM7, do not exhibit profound sensitivity to PARPi that might be associated with a BRCAness phenotype.

## Results

### Parallel chemosensitivity screens in OS and non-OS tumour cell lines

**Figure 1:**
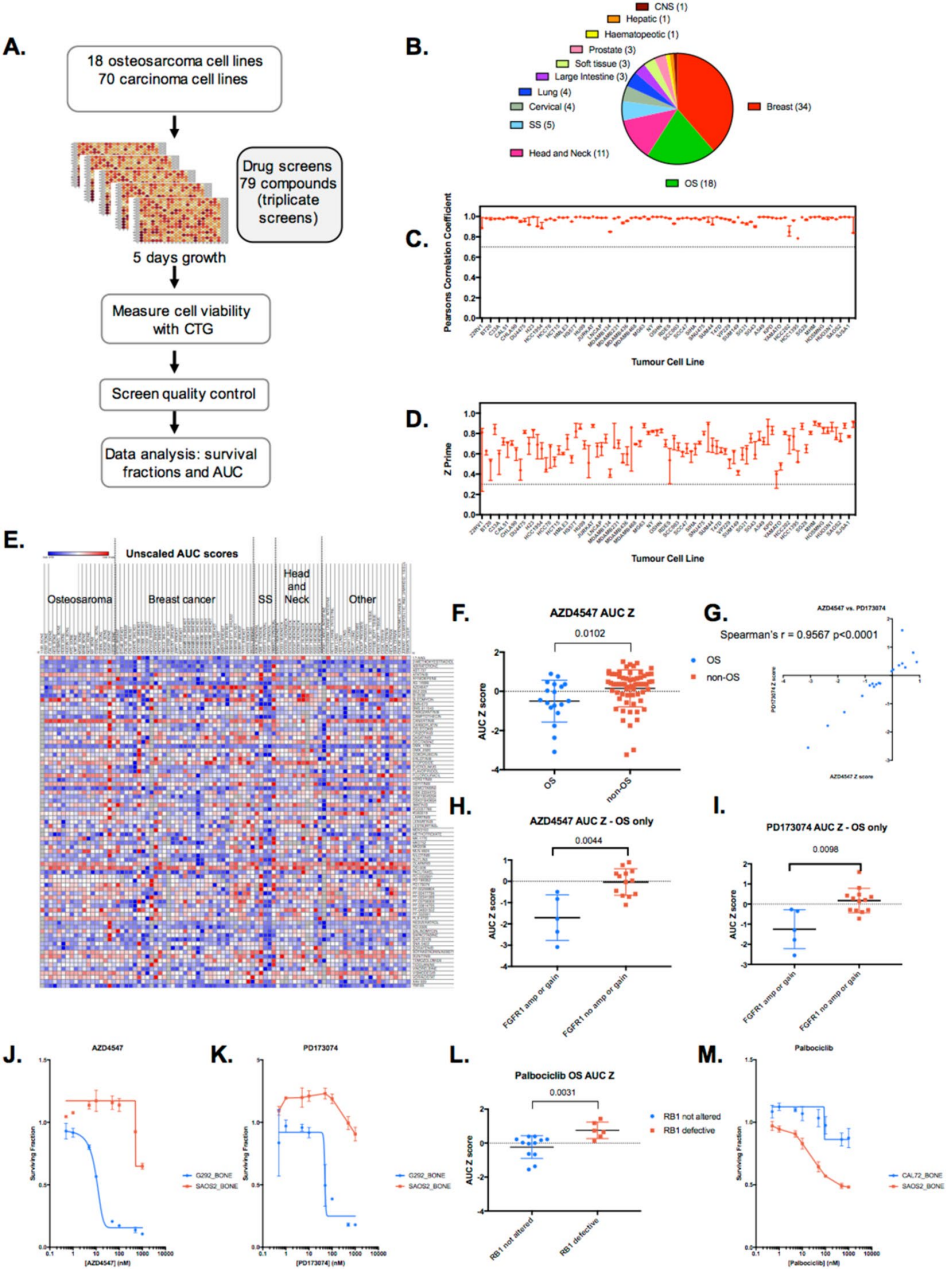
Drug screening overview. (**A)** Schematic of high-throughput small molecule chemosensitivity screens. Tumour cell lines (TCLs) were plated in 384-well plates in log phase and exposed to small molecule inhibitors for five continuous days, at which point cell viability was estimated by use of CellTitreGlo reagent (CTG). (**B**) Piechart illustrating the histotypes of the 88 tumour cell lines included in the study. (**C**) Scatter plot of Pearson’s correlation values between triplicate screens. Only tumour cell lines that had Pearson’s correlation values between triplicate screens of >0.7 were included in the final dataset. Median and range shown. (**D**) Scatter plot of Z prime values for each replica of the screen. Only tumour cell lines that had median Z prime values of >0.3 were included in the final dataset. Median and range shown. (**E**) Heatmap illustrating Area Under the Curve (AUC) values for each drug in each tumour cell line. Heatmaps illustrating the dataset described as scaled AUC scores and unscaled AUC Z scores are show in Supplementary Figure 1. (**F**) Box whiskers plot of AZD4547 FGFR inhibitor AUC Z scores in 88 tumour cell lines, illustrating the relative sensitivity of OS TCLs. p value 0.0102, Mann-Whitney test. (**G**) Scatter plot of AZD4547 FGFR inhibitor AUC Z scores in OS TCLs, compared to AUC Z scores for the FGFR inhibitor PD173074. (**H**) Box whiskers plot of AZD4547 FGFR inhibitor AUC Z scores in OS tumour cell lines classified according to *FGFR1* gene status. p value 0.0044, Mann-Whitney test. (**I**) Box whiskers plot of PD173074 FGFR inhibitor AUC Z scores in OS tumour cell lines classified according to *FGFR1* gene status. p value 0.0098, Mann-Whitney test. (**J**,**K**) Example dose response survival curves derived from screen data for FGFR inhibitors in two OS TCLs, G292 (*FGFR1* amplified) and SAOS2 (*FGFR1* copy number neutral). (**L**) Box whiskers plot of Palbociclib CDK4/6 inhibitor AUC Z scores in OS tumour cell lines classified according to *Rb* status. p value 0.0031, Mann-Whitney test. (**M**) Example dose response survival curves derived from screen data for palbociclib in two OS TCLs, CAL72 (Rb defective) and SAOS2 (Rb not altered).

In order to generate a resource that could be used as a starting point to investigate sensitivity to targeted agents in OS, we compiled a panel of 18 well-characterised OS tumour cell lines (TCLs) and subjected these high-throughput drug sensitivity screening, alongside 70 tumour cell lines derived from the following carcinomas: breast (34) head and neck (11), synovial sarcoma (5), cervical (4), lung (4), large intestine (3), prostate (3), soft tissue (other than synovial sarcoma) (3), haematopoietic (1), central nervous system (CNS) (1), and hepatic (1) (Fig. 1A and listed in Supplementary Table 1). Given the interest in PARP inhibitor sensitivity in OS, we also included in the tumour cell line panel a PARP inhibitor sensitive breast tumour cell line, SUM149, with loss of function *BRCA1* mutation (BRCA1 p.P724fs*12) as well as a PARP inhibitor resistant SUM149-subclone, SUM149.B1*.S, that has partially restored BRCA1 function because of a CRISPR-Cas9 mutagenesis-engineered *BRCA1* intragenic reversion mutation^8^. This *BRCA1* reversion mutation restores the native open reading frame of the *BRCA1* gene, recapitulates RAD51 nuclear localisation (a key molecular event in BRCA1-mediated DNA repair) and causes profound PARP inhibitor resistance^8^.

For chemosensitivity screening, we used an in–house curated small molecule library of 79 small-molecule inhibitors that are either in clinical use for the treatment of cancer or in late-stage clinical development (Supplementary Table 2). To maximise the possibility of identifying chemosensitivity effects, each compound was included in the screening library at eight different concentrations. Tumour cell lines were plated in 384-well-plates and then 24 hours later exposed to library drugs; cells were then continuously cultured for a further five days in the presence of drug, at which point we estimated cell viability in each well by the use of CellTitreGlo reagent. Each cell line was screened in triplicate and we combined data from replica screens in the final analysis. After processing the data from each plate to account for plate to plate variation across the library, we converted CellTitreGlo readings into: (i) surviving fraction (SF) data for triplicate drug sensitivity screens in 88 tumour cell lines (Supplementary Table 3); (ii) unscaled area under the curve (Supplementary Table 4); (iii) scaled AUC scores calculated as in^9^ (Supplementary Table 5); and (iv) a Z score normalised version of the unscaled AUC values (Supplementary Table 6 and Supplementary Figure 1A,B). These datasets, provided as a resource in the Supplementary Tables, allowed us to compare drug sensitivity effects between different tumour cell lines.

Prior to assessing the drug sensitivity effects in tumour cell lines, we first assessed the reproducibility of replica screens and the dynamic range found in each individual screen. We estimated the reproducibility of triplicate screens in each cell line by calculating Pearson’s correlation values between replicas and estimated dynamic range by calculating Z′ values for each screen (Fig. 1C,D). For the final resource (Fig. 1E, Supplementary Tables), we only included data where Pearson’s correlation values between replicas were >0.7 and median Z′ values were >0.3 (Fig. 1C,D).

### Drug sensitivity effects in OS tumour cell lines

Amplification of the Fibroblast Growth Factor Receptor 1 (*FGFR1*) gene in OS tumour cell lines has previously been shown to predict enhanced sensitivity to FGFR inhibition by the pan-FGFR inhibitor NVP-BGJ398^10^. To assess the ability of our screen data to identify this known chemosensitivity effect, we interrogated the screen data from OS tumour cell lines for two different FGFR inhibitors, AZ4547 and PD173074. We noted that, in general, OS TCLs exhibited greater sensitivity to AZD4547 (Fig. 1F, p = 0.01, Mann-Whitney uncorrected test), as previously noted^11^ and found that AZ4547 *vs*. PD173074 sensitivities in OS TCLs were highly correlated (Fig. 1G, Spearman’s r = 0.96, p < 0.0001, uncorrected test). We also found that OS tumour cell lines with *FGFR1* amplification (G292, NOS1 and CAL72) or *FGFR1* gain (HU09 and NY) were significantly more sensitive to both AZ547 and PD173074 than those OS tumour cell lines without *FGFR1* copy number alterations (Fig. 1H,I, p < 0.01 in both cases, Mann-Whitney uncorrected test and Fig. 1J,K). Similarly, we noticed that, as for other cancer histotypes^12^, RB1 (Retinoblastoma) tumour suppressor defects in OS TCLs (Supplementary Table 7^11^ were associated with resistance to the CDK4,6 inhibitor, palbociclib (Fig. 1L, p 0.031, Mann-Whitney uncorrected test), suggesting that genotype-specific drug sensitivity effects in OS TCLs could be identified.

### PARP inhibitor sensitivity in OS tumour cell lines

Based on these proof of concept examples, we interrogated the dataset to examine the sensitivity of OS TCLs to PARP inhibitors. Recent analysis of exome DNA resequencing data from treatment naïve OS has suggested that a significant fraction of OS might exhibit a pattern of chromosomal aberrations, specifically chromosomal loss of heterozygosity (LOH) effects, that are somewhat reminiscent of mutational patterns seen in tumours from cancer patients with germ-line *BRCA1* or *BRCA2* gene mutations^5^. These characteristics of BRCAness might have therapeutic implications, as the DNA repair defects thought to lead to this particular type of LOH effect, can often cause sensitivity to PARPi.

**Figure 2:**
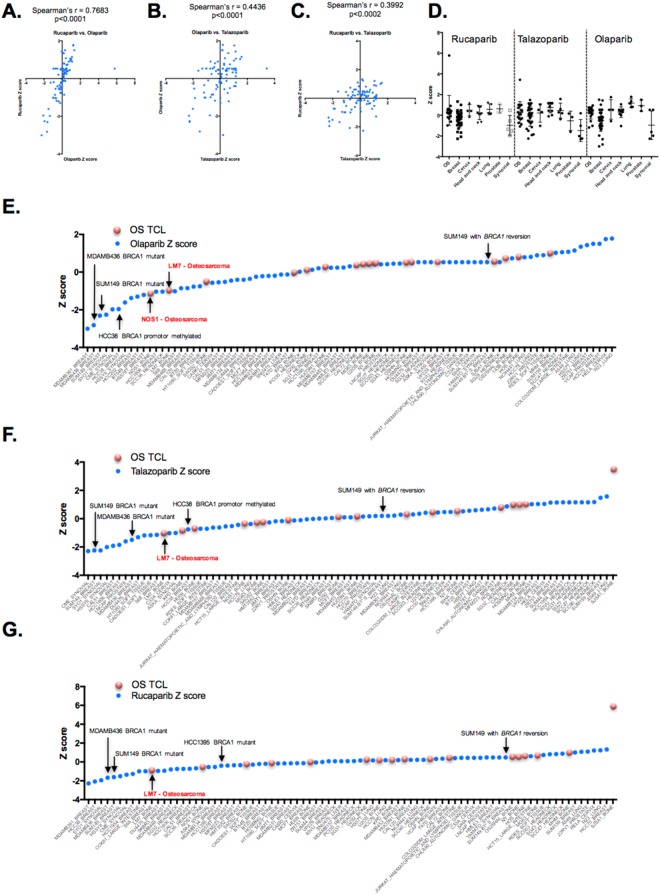
PARP inhibitor sensitivity in tumour cell lines determined by the chemosensitivity screen. (**A–C**) Scatter plots illustrating the correlation in AUC Z scores for three different PARP inhibitors (rucaparib, talazoparib, olaparib) in 88 tumour cell lines. For each plot, the Spearman’s correlation coefficient, r, is shown. (**D**) Scatter plots illustrating AUC Z scores for rucaparib, talazoparib and olaparib classified according to tumour cell line histotype. Each dot represents a single TCL; error bars represent mean and standard error of the mean. (**E**–**G)** Waterfall plot illustrating olaparib (**E**), talazoparib (**F**) and rucaparib (**G**) AUC Z scores in 88 TCLs. Breast tumour cell lines with *BRCA1* alterations are shown, as is the PARP inhibitor resistant/*BRCA1* revertant SUM149-subclone (“SUM149 with BRCA1 reversion”, see main text). Red dots indicate OS tumour cell lines, blue dots indicate non-OS TCLs.

To estimate the relative PARPi sensitivity of OS (and other histology) TCLs, we extracted from the dataset the AUC Z scores for three clinical PARP inhibitors; rucaparib, olaparib and talazoparib. When we compared the sensitivity profiles of TCLs to the three different PARP inhibitors, we found these to be highly correlated (Fig. 2A–C). When assessing the relative sensitivity of cell line models from different cancer histologies, we found that in general, breast tumour cell lines and also synovial sarcoma tumour cell lines displayed the greatest level of PARP inhibitor sensitivity (Fig. 2D). Indeed, synovial sarcoma TCLs not only showed PARP inhibitor sensitivity, as previously described^13^, but also enhanced sensitivity to the DNA damaging agents doxorubicin, gemcitabine and camptothecin (Supplementary Figure 1). As positive and negative controls respectively, we included in the screen *BRCA1* mutant, PARPi sensitive SUM149 cells and a PARPi resistant SUM149 subclone with a *BRCA1* reversion mutation, described elsewhere^8^. As expected, we found SUM149 cells to display one of the most profound PARP inhibitor sensitivity effects in the panel of 92 tumour cell lines (ranked 3rd, 2nd, and 5th for olaparib, talazoparib and rucaparib sensitivity respectively, Fig. 2E–G), whereas the SUM149 *BRCA1* revertant tumour clone displayed profound PARPi resistance (Fig. 2E–G). We also noted additional *BRCA1* mutant tumour cell lines (MDAMB436, HCC1395) and a *BRCA1* promoter hypermethylated tumour cell line HCC38^14^ to display PARPi sensitivity, as did a number of synovial sarcoma tumour cell lines (SYO1, CME, HS-SYII, ASKA), as described earlier (Fig. 2E–G).

OS TCLs demonstrated a spectrum of PARPi sensitivity effects (Fig. 2E–G). Out of 18 OS tumour cell lines assessed, only LM7 demonstrated profound sensitivity to PARPi of a scale similar to that seen in *BRCA1* mutant SUM149 cells (Fig. 2E–G). LM7 cells were originally derived from SAOS2 cells by repeated passage of SAOS2 through the lungs of nude mice^15^. We found SAOS2 cells to be relatively resistant to all three PARPi, when compared to LM7 (Fig. 2E–G), suggesting that heightened PARPi sensitivity in LM7 was an acquired phenotype. We also assessed the PARPi sensitivity of OS TCLs proposed to have genomic signatures indicative of BRCAness; MG63, SAOS2, and HOSMNNG^7^. We found each of these TCLs to be relatively resistant to PARPi, when compared to SUM149 cells (median AUC for olaparib, Rucaparib and Talazoparib for MG63, SAOS2, and HOSMNNG being 0.985, 0.896, 0.967, compared to 0.722 in SUM149) and to display a similar level of PARPi resistance as the *BRCA1* revertant SUM149 subclone (Fig. 2E–G).

**Figure 3:**
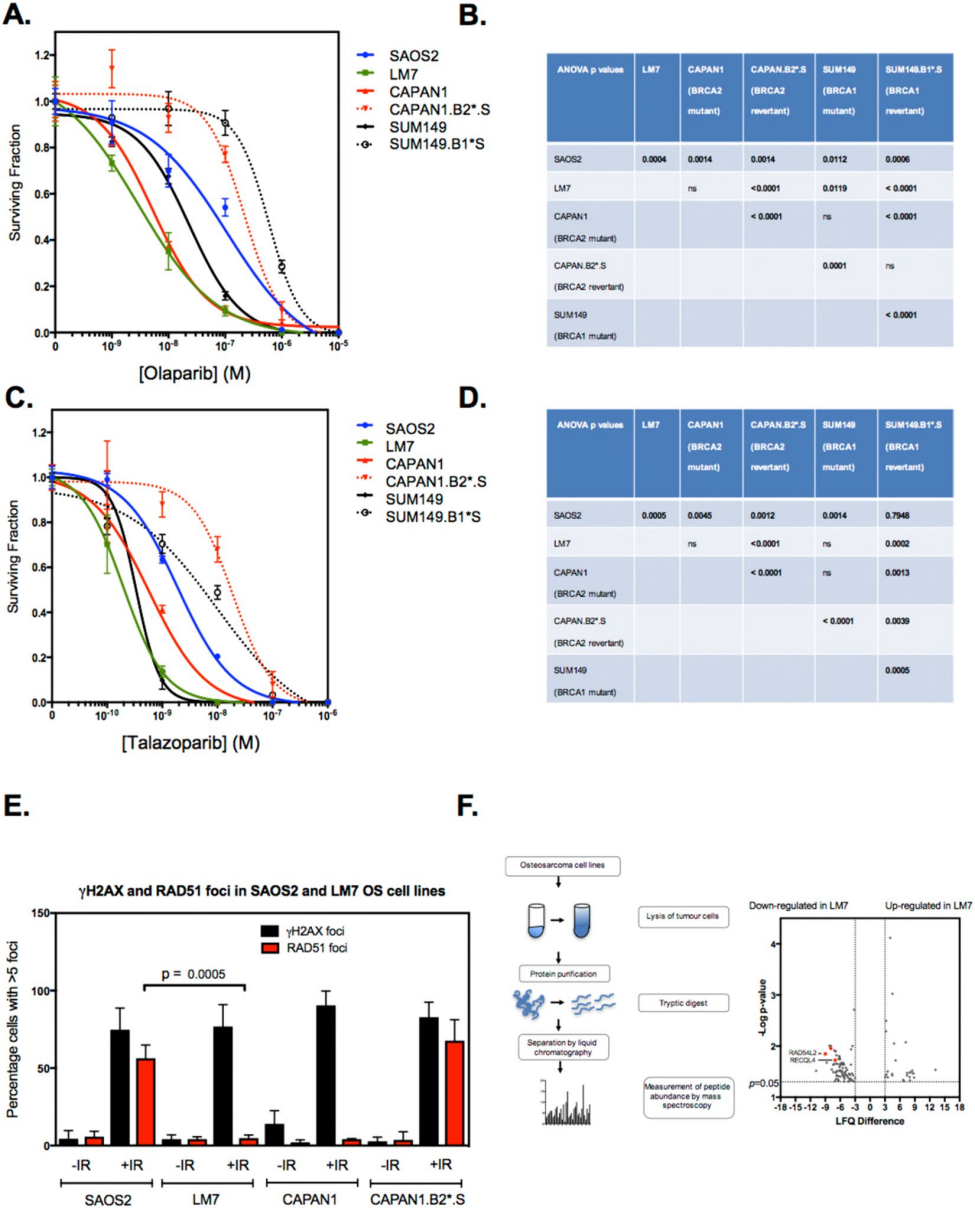
PARP inhibitor sensitivity and RAD51 defects in OS tumour cell lines. (**A**) Olaparib dose response curves derived from clonogenic assays where TCLs were exposed to PARP inhibitor for 14 continuous days. LM7 and SAOS2 cells were compared to CAPAN1 cells (*BRCA2* mutant, PARP inhibitor sensitive), CAPAN1.B2*.S (*BRCA2* revertant, PARP inhibitor resistant), SUM149 (*BRCA1* mutant, PARP inhibitor sensitive) and SUM149.B1*.S (*BRCA1* revertant, PARP inhibitor resistant) cells. Error bars represent SEM from triplicate experiments. (**B)** Table illustrating ANOVA *p* values from olaparib sensitivity comparisons in (**A**). (**C**,**D)** Talazoparib dose response curves and ANOVA *p* values derived from clonogenic assays where TCLs were exposed to PARP inhibitor for 14 continuous days. As for (**A**,**B**) (**E**) Bar chart illustrating the quantitation of nuclear RAD51 foci in tumour cells after exposure to ionising radiation. Cells were plated in triplicate in 6-well plates on coverslips. After 24 hours, tumour cells were irradiated (10 Gy) and cultured for a subsequent four hours, at which point cells were fixed and immunostained. Confocal microscopy was used to visualise and score nuclear γH2AX and RAD51 foci. Cells containing more than five nuclear foci were considered positive. Mean ± SEM for three independent experiments are shown. LM7 cells exhibited significantly decreased nuclear RAD51 foci formation compared to SAOS2 (*p* = 0.0005). *p* values were calculated using Student’s t test. (**F**) Differential protein levels in SAOS2 and LM7 cells. Schematic of experimental procedure used to determine proteomic profiles in LM7 and parental SAOS2 cells is shown left. Following lysis, protein purification, and tryptic digest, peptides were separated by liquid chromatography and measured by mass spectrometer. Label-free proteome quantification was performed using the MaxQuant software environment to determine the quantitative abundance of 6696 peptides with a false discovery rate of less than one percent. Volcano plot of median difference in proteomic abundance between LM7 and SAOS2 shown right. RAD54L2 and RECQL4 are highlighted. Only peptides with a significant difference in abundance between LM7 and SAOS2 are shown (p < 0.05 effects (two sided heteroscedastic t-test) with an absolute difference in log2(LFQ) greater than 3).

To investigate the PARPi sensitivity phenotype of LM7 cells in more detail, we used two-week clonogenic drug exposure assays to confirm the results identified in the screen. Although exome sequencing of LM7 cells did not identify *BRCA1* nor *BRCA2* gene alterations, LM7 cells exhibited comparable olaparib sensitivity to BRCA2 defective (*BRCA2* c.6774delT truncating mutant) CAPAN1 pancreatic ductal carcinoma cells^16^ (ANOVA p = 0.3757) but significantly greater sensitivity to olaparib than *BRCA1* mutant SUM149 cells (ANOVA p = 0.0119), the *BRCA1* revertant SUM149 subclone, and a previously validated PARPi resistant *BRCA2* revertant CAPAN1 subclone, CAPAN1.B2*.S^8^ (ANOVA p < 0.0001, Fig. 3A,B). LM7 cells also exhibited similar sensitivity to Talazoparib, demonstrating significantly greater sensitivity to this PARPi than CAPAN1.B2*.S and SUM149.B1*.S (ANOVA p < 0.001, Fig. 3C,D).

### LM7 cells exhibit defects in RAD51 and other proteins associated with homologous recombination

The nuclear localisation of the DNA recombinase RAD51 to the site of DNA damage is a key step in the DNA repair processes mediated by BRCA1 and BRCA2 and is critical in repairing the DNA lesions caused by PARP inhibitors. This nuclear localisation of RAD51 can be estimated by microscopy-based imaging of discrete nuclear RAD51 foci^17^. We investigated whether LM7 cells exhibited a RAD51 nuclear localisation defect, finding that in response to DNA damage, LM7 cells exhibited significantly decreased nuclear RAD51 foci formation, compared to SAOS2 cells (p = 0.0005, Student’s t test, Fig. 3E), an observation consistent with their PARPi sensitivity (Fig. 3A–D). Although we were not able to detect DNA mutations in genes known to control PARPi sensitivity in LM7 cells (data not shown) proteomic profiling of SAOS2 and LM7 cells identified differentially expressed proteins that might explain the PARPi sensitivity phenotype in LM7 cells (Supplementary Table 8); in totality, we identified 26 proteins that were overexpressed in LM7 compared to SAOS2 and 80 proteins that were under expressed in LM7 (Fig. 3F). These included two proteins previously linked to DNA repair by homologous recombination; RAD54L2 (ranked 2^nd^ most under expressed in LM7 *vs*. SAOS2, p = 0.01, two sided heteroscedastic t-test) and RECQL4^18^ (ranked 13^th^ most under expressed in LM7 tumour cells, p = 0.019, two sided heteroscedastic t-test).

## Discussion

We describe here chemosensitivity profiles for a panel of OS tumour cell lines. Our intention in carrying out this work was to generate a reference dataset that could be used for a variety of applications, ranging from the selection of tumour cell models with defined drug sensitivity/resistance phenotypes for subsequent experiments, through to the identification of drug sensitivity effects associated with molecular features found in these diseases. Given the “resource” nature of the work, we have included within this report, both raw and processed data so that subsequent analyses can be facilitated. For example, as the molecular profiling of these tumour cell line models is enhanced, for example by the addition of proteomic profiles of OS tumour cell lines, the utility of the chemosensitivity profiles we describe here will no doubt be enhanced.

As part of this work, we highlight how the dataset can be used to assess PARP inhibitor sensitivity in OS tumour cell lines. By comparing the PARP inhibitor sensitivity of OS tumour cell lines to the scale of sensitivity in breast tumour cell lines with BRCA1 defects and also isogenic tumour cell models with a well-established and clinically relevant mechanism of PARP inhibitor resistance, we have been able to make a comparative analysis that suggests that whilst most OS tumour cell lines do not exhibit the exquisite level of PARP inhibitor sensitivity seen in *BRCA1* mutant breast tumour cell lines, the LM7 model does display evidence of BRCAness and a PARP inhibitor sensitivity phenotype. Although this is a singular model, the LM7 cell line therefore does provide a reagent for investigating mechanisms that cause PARP inhibitor sensitivity in OS.

It is important to describe the limitations of the resource we provide. Using three-dimensional tissue culture approaches would assess whether the drug sensitivity and resistance phenotypes we see here are robust in the face of the loss of an adherent surface or changes in cell morphology and organisation often more apparent in three dimensional, as opposed to two-dimensional culture systems. We also note that more prolonged drug exposures, beyond the five-day drug exposure used here, might also elicit distinct effects, especially for agents whose cell inhibitory properties are amplified by drug exposure over multiple cell cycles. Nevertheless, we did find that the five-day drug exposure used was sufficient to document differences in PARP inhibitor sensitivity between *BRCA1* mutant and *BRCA1* revertant clones; the synthetic lethal effects of PARP inhibitors in *in vitro* cell culture often require drug exposure over several cell cycles (Farmer *et al*., 2005), but the five-day exposure appears sufficient here.

## Materials and Methods

### Cell lines

The origin of non-OS tumour cell lines is described in (Campbell *et al*., 2016; Jones *et al*., 2017). For OS tumour cell lines, G292 (clone A141B1), MG63, CAL72, HU03N1, OSA/SJSA-1, NOS1, SAOS2, U2OS, HU09, NY and HOS were gifts from Ultan McDermott at the Wellcome Trust Sanger Institute (WTSI); LM7, OHSN, OSH25HAL (HAL) MHM, and KPD were gifts from Ola Myklebost, Univeristy of Bergen, Norway. Head and neck cancer cell line models were obtained from Susanne Gollin and Theresa Whiteside (University of Pittsburgh), Tom Carey (University of Michigan) and Hans Joenje (VU Medical Centre, NL). All cell lines were maintained as per the suppliers’ instructions. STR typing of 10 loci was performed on each cell line using the GenePrint 10 system (Promega) and used to confirm the identity of cell lines prior to storage.

### Small molecule inhibitor high-throughput screens

Small molecules were purchased as solid from suppliers listed in Supplementary Table 2 and stored in DMSO. Prior to 384 well-plate screens, solid small molecules were resuspended in DMSO as 10 mM stocks, prior to further dilution in DMSO to create 384 well-plates containing a titration (0.5, 1, 5, 10, 50, 100, 500, 1000 nM). A Hamilton Microlab Star liquid handling platform was used for this and all subsequent liquid handling steps with the exception of cell seeding.

Cells growing in log phase were seeded in 384 well-plates at 250–500 cells per well in 50 uL of tissue culture media using a Thermo Fisher Multi-Drop Combi. Cells were initially plated at a density to ensure that each cell line was in growth phase by the end of the five-day treatment, similar to^19^. 24 hours after seeding, media was removed and changed to media containing the small molecule inhibitor library, as detailed above. Cells were then continuously cultured in the presence of small molecule inhibitors for a period of five days, at which point cell viability was estimated by adding 20 uL (diluted 1/4 in PBS) Cell-Titre Glo (Promega) to media. After 10 minutes incubation at room temperature, Cell-Titre Glo generated luminescence was captured using a Victor X-Light plate reader. Luminescence values from each well were normalised to the median of signals from wells exposed to DMSO only (no small molecule inhibitor) to generate surviving fractions (SF). In total, each tumour cell line was screened three times, generating triplicate SF data sets (Supplementary Table 3). Surviving fractions were then used to plot dose-response survival curves, generated using 3-parameter logistic regression analysis via the *drc* R-package^20^. Using *drc*, Area under the curve (AUC) values were calculated from dose-response survival curves (Supplementary Table 4, unscaled AUC scores for 88 tumour cell lines). These unscaled AUC values (calculated as per Activity Area A_ref_ = 0 values in^9^ were then scaled according to A_ref_ = *max* (0, A_low_) (as^9^) where A_low_ is the activity at the lowest concentration of drug used, up to the maximum tested concentration (Supplementary Table 5, scaled AUC scores for 88 tumour cell lines). Unscaled AUC values for each drug in 88 tumour cell lines were also standardized, generating robust Z scores based upon the median AUC effect in all 88 tumour cell lines and the median absolute mediation of these effects (Supplementary Table 5 – Z normalised unscaled AUC scores for 88 tumour cell lines). To compare drug sensitivities to FGFR1 status in OS TCLs, the cBioPortal was used to access Comparative Genomic Hybridisation (CGH) data for OS TCLs from the Broad Institute CCLE, scored via the GISTIC algorithm to define amplification (score +2) of *FGFR1*. Rb status (Supplementary Table 7) in OS TCLs from^11^ was used to assess the correlation between Rb status and palbociclib sensitivity. PARP inhibitor sensitivity effects identified in the high-throughput screen were confirmed using clonogenic survival assays, performed as described in^21^.

### Confocal microscopy

Nuclear RAD51 foci were visualised and quantified by immunohistochemistry as described previously^8^.

### Proteomic profiling

Proteomic abundance for the osteosarcoma tumour cell lines was performed as follows: Following lysis, protein purification, and tryptic digest, peptides were separated by liquid chromatography and measured mass spectrometer. Label-free proteome quantification was performed using the MaxQuant software environment^22,23^ to determine the quantitative abundance of 6696 peptides with a false discovery rate of less than one percent. Log2 transformed label free quantification (LFQ) values were used for all analyses. Proteins were identified as differentially expressed if they were associated with a p < 0.05 effect (two sided heteroscedastic t-test) and an absolute difference in log2(LFQ) greater than 3.

### Data availability

All processed data is present in Supplementary Tables 1–8. Raw luminescence data from the chemosensitivity screen is available upon request.

## Electronic supplementary material

Supplementary Information

Supplementary Table 1

Supplementary Table 2

Supplementary Table 3

Supplementary Table 4

Supplementary Table 5

Supplementary Table 6

Supplementary Table 7

Supplementary Table 8
