# Author Correction: Chemosensitivity profiling of osteosarcoma tumour cell lines identifies a model of BRCAness

**DOI:** 10.1038/s41598-018-30922-8

**Published:** 2018-08-21

**Authors:** Harriett Holme, Aditi Gulati, Rachel Brough, Emmy D. G. Fleuren, Ilirjana Bajrami, James Campbell, Irene Y. Chong, Sara Costa-Cabral, Richard Elliott, Tim Fenton, Jessica Frankum, Samuel E. Jones, Malini Menon, Rowan Miller, Helen N. Pemberton, Sophie Postel-Vinay, Rumana Rafiq, Joanna L. Selfe, Alex von Kriegsheim, Amaya Garcia Munoz, Javier Rodriguez, Janet Shipley, Winette T. A. van der Graaf, Chris T. Williamson, Colm J. Ryan, Stephen Pettitt, Alan Ashworth, Sandra J. Strauss, Christopher J. Lord

**Affiliations:** 1 The CRUK Gene Function Laboratory and Breast Cancer Now Toby Robins Research Centre, The Institute of Cancer Research, London, SW3 6JB UK; 2 UCL Cancer Institute, University College London, London, WC1E 6DD UK; 3 Clinical and Translational Sarcoma Research, The Institute of Cancer Research, London, SM2 5NG UK; 4 The Royal Marsden NHS Foundation Trust, Fulham Road, London, SW3 6JJ UK; 5 School of Biosciences, University of Kent, Canterbury, Kent, CT2 7NJ UK; 6 Sarcoma Molecular Pathology Laboratory, The Institute of Cancer Research, London, SM2 5NG UK; 7 Edinburgh Cancer Research Centre, IGMM, University of Edinburgh, Edinburgh, EH4 2XR UK; 8 Systems Biology Ireland, University College Dublin, Dublin 4, Ireland; 9 Present Address: UCSF Helen Diller Family Comprehensive Cancer Centre, San Francisco, California, 94158 USA

Correction to: *Scientific Reports* 10.1038/s41598-018-29043-z, published online 13 July 2018

In the original version of this Article, Tim Fenton was incorrectly affiliated with ‘UCL Cancer Institute, University College London, London, WC1E 6DD, UK’. The correct affiliation is listed below.

School of Biosciences, University of Kent, Canterbury, Kent. CT2 7NJ

This error has now been corrected in the PDF and HTML versions of the Article and in the Supplementary Information.

