# Supplementary material for: Chemosensitivity profiling of osteosarcoma tumour cell lines identifies a model of BRCAness
Source: Sci Rep. 2018 Jul 13;8:10614. doi: 10.1038/s41598-018-29043-z (PMC6045584; doi:10.1038/s41598-018-29043-z)
Supplement: Supplementary file 1 — Supplementary Information [file 41598_2018_29043_MOESM1_ESM.docx]

**Chemosensitivity profiling of osteosarcoma tumour cell lines identifies a model of BRCAness**

Harriett Holme*^1,2^, Aditi Gulati*^1^, Rachel Brough*^1^, Emmy D.G. Fleuren^1,3^, Ilirjana Bajrami^1^, James Campbell^1^, Irene Y. Chong^1,4^, Sara Costa-Cabral^1^, Richard Elliott^1^, Tim Fenton^5^, Jessica Frankum^1^, Samuel E. Jones^1^, Malini Menon^1^, Rowan Miller^1^, Helen N. Pemberton^1^, Sophie Postel-Vinay^1^, Rumana Rafiq^1^, Joanna L. Selfe^6^, Alex von Kriegsheim^7^, Amaya Garcia Munoz^8^, Javier Rodriguez^8^, Janet Shipley^6^, Winette T.A. van der Graaf^3^, Chris T. Williamson^1^, Colm J. Ryan^8^, Stephen Pettitt^1^, Alan Ashworth^1,9*^, Sandra J. Strauss^2*^ and Christopher J. Lord^1*^

**Supplementary Information**


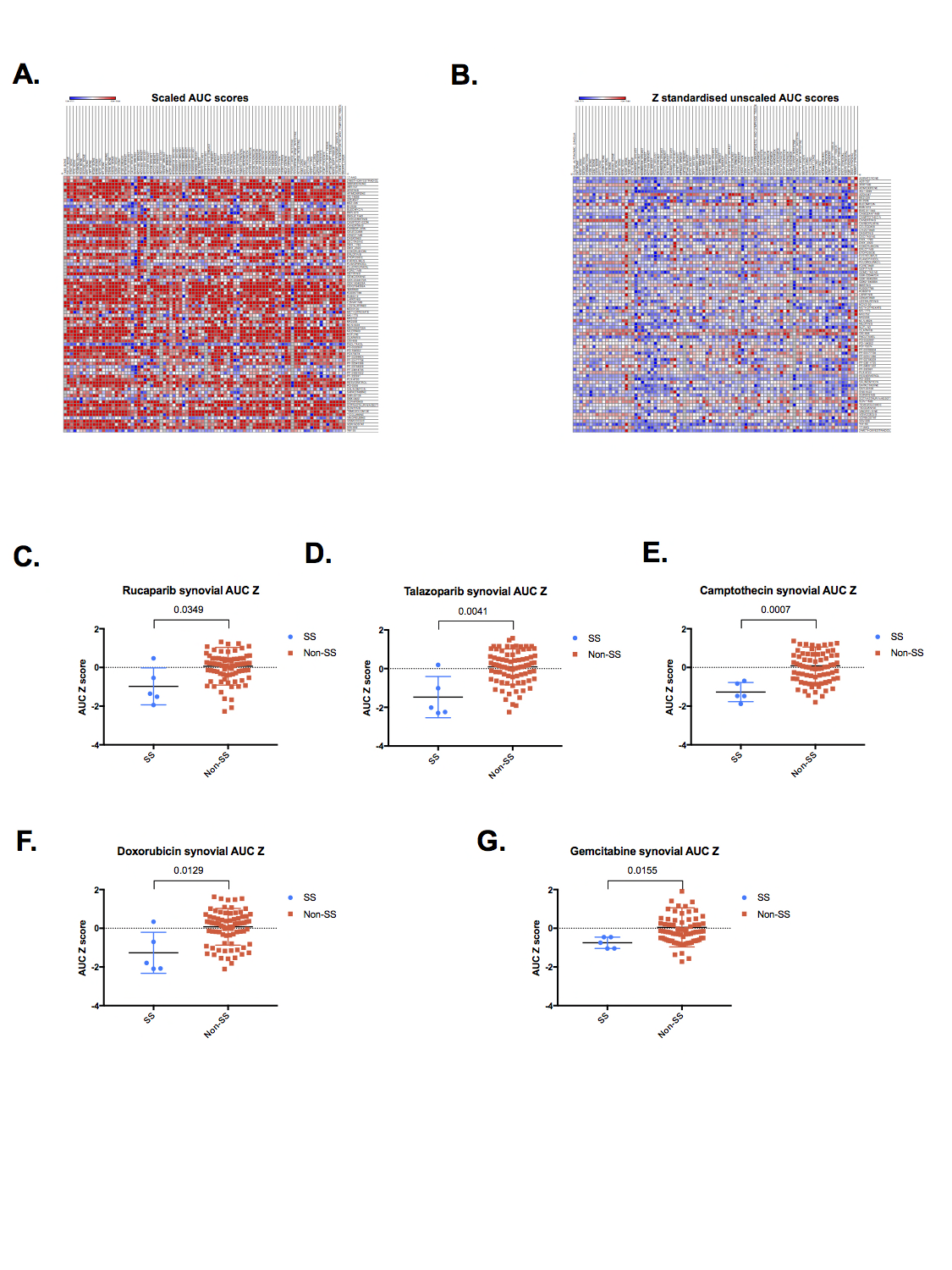


**Supplementary Figure 1. A.** Scaled AUC scores in 88 tumour cell lines. **B.** Z score standardized unscaled AUC scores in 88 tumour cell lines. **C-G.** Box whiskers plots of DNA damaging agents in 88 tumour cell lines, illustrating the relative sensitivity of synovial sarcoma TCLs. *p* values shown calculated using Mann-Whitney test.

**Supplementary Table 1.** Tumour cell lines used in the study

**Supplementary Table 2:** Small molecule inhibitors used in the study

**Supplementary Table 3:** Surviving fraction data for triplicate drug sensitivity screens in 88 tumour cell lines

**Supplementary Table 4:** Unscaled AUC scores for 88 tumour cell lines

**Supplementary Table 5:** Scaled AUC scores for 88 tumour cell lines

**Supplementary Table 6:** Z-normalised unscaled AUC scores for 88 tumour cell lines

**Supplementary Table 7:** Rb annotation for OS TCLs

**Supplementary Table 8:** Quantitative abundance of 6696 peptides with FDR <1% detected by mass spec. profiling in LM7 and SAOS2 cells
